# Quantification correction for free-breathing myocardial T_1ρ_ mapping in mice using a recursively derived description of a T_1ρ_* relaxation pathway

**DOI:** 10.1186/s12968-022-00864-2

**Published:** 2022-05-09

**Authors:** Maximilian Gram, Daniel Gensler, Petra Albertova, Fabian Tobias Gutjahr, Kolja Lau, Paula-Anahi Arias-Loza, Peter Michael Jakob, Peter Nordbeck

**Affiliations:** 1 Department of Internal Medicine I, University Hospital Würzburg, Würzburg, Germany; 2 Experimental Physics 5, University of Würzburg, Würzburg, Germany; 3 Comprehensive Heart Failure Center (CHFC), University Hospital Würzburg, Würzburg, Germany; 4 Department of Nuclear Medicine, University Hospital Würzburg, Würzburg, Germany

**Keywords:** T1rho, T1ρ, Spin-lock, Mapping, Quantitative MRI, Correction, Cardiac, Radial

## Abstract

**Background:**

Fast and accurate T_1ρ_ mapping in myocardium is still a major challenge, particularly in small animal models. The complex sequence design owing to electrocardiogram and respiratory gating leads to quantification errors in in vivo experiments, due to variations of the T_1ρ_ relaxation pathway. In this study, we present an improved quantification method for T_1ρ_ using a newly derived formalism of a T_1ρ_* relaxation pathway.

**Methods:**

The new signal equation was derived by solving a recursion problem for spin-lock prepared fast gradient echo readouts. Based on Bloch simulations, we compared quantification errors using the common monoexponential model and our corrected model. The method was validated in phantom experiments and tested in vivo for myocardial T_1ρ_ mapping in mice. Here, the impact of the breath dependent spin recovery time T_rec_ on the quantification results was examined in detail.

**Results:**

Simulations indicate that a correction is necessary, since systematically underestimated values are measured under in vivo conditions. In the phantom study, the mean quantification error could be reduced from − 7.4% to − 0.97%. In vivo, a correlation of uncorrected T_1ρ_ with the respiratory cycle was observed. Using the newly derived correction method, this correlation was significantly reduced from r = 0.708 (p < 0.001) to r = 0.204 and the standard deviation of left ventricular T_1ρ_ values in different animals was reduced by at least 39%.

**Conclusion:**

The suggested quantification formalism enables fast and precise myocardial T_1ρ_ quantification for small animals during free breathing and can improve the comparability of study results. Our new technique offers a reasonable tool for assessing myocardial diseases, since pathologies that cause a change in heart or breathing rates do not lead to systematic misinterpretations. Besides, the derived signal equation can be used for sequence optimization or for subsequent correction of prior study results.

**Supplementary Information:**

The online version contains supplementary material available at 10.1186/s12968-022-00864-2.

## Introduction

Cardiovascular diseases are the leading cause of death worldwide, due to population growth and an aging population [1, 2]. Cardiomyocyte necrosis, as well as the formation of fibrosis and collagen i.e. after myocardial infarction (MI), can cause harmful remodeling of the myocardium, ultimately leading to heart failure [3–6]. At cellular level, cell death is followed by an increase in extracellular space, which affects the interaction between free water and macromolecules [7, 8]. Applying advanced imaging tools in both animal models as well as patient care has led to greatly increased knowledge [9–11], proving that even subtle scar formation has important diagnostic, prognostic and therapeutic clinical implications [12]. Therefore, the development of even more sensitive tools for detailed tissue characterization is of great importance.

Cardiovascular magnetic resonance (CMR) offers various non-invasive diagnostic options for investigating the dynamic processes of remodeling in vascular and cardiac disease [13–15]. Late gadolinium enhancement (LGE) represents the current gold standard for the detection of fibrotic scars, as it offers high contrast between the infarcted zone and the surrounding healthy myocardial tissue. However, this technique requires the administration of gadolinium based contrast agent, which is contraindicated in certain patients, particularly those with reduced renal function [16]. Over the past decade, endogenous imaging techniques based on the quantification of relaxation times have been proposed to address this problem [17]. Here, the rotating frame relaxation times show promising results, since in comparison to the convectional spin–lattice (T_1_) and spin–spin (T_2_) relaxation a high degree of sensitivity for the slow tumbling regime can be generated and thus slow proton exchange and water-macromolecule interactions dominate the relaxation process [18–20]. By convention, the longitudinal rotating frame relaxation T_1ρ_ relates to relaxation processes along an on-resonant radiofrequency field that is applied in the form of a spin-lock (SL) pulse [21–23]. Off-resonant SL techniques (${\mathrm{T}}_{1\uprho }^{\mathrm{off}}$) and relaxation along fictitious fields in the n-th rotating frame (T_RAFFn_) have also been suggested for the generation of specific tissue contrasts [24–27].

In the context of clinical CMR, T_1ρ_-based imaging has already been applied successfully in several studies [28, 29]. T_1ρ_ was verified as a sensitive marker for the detection and characterization of tissue damage caused by ischemia and subsequent myocardial damage [30–32]. Yet, the main advantage of T_1ρ_ -based imaging might be the selective sensitivity of the relaxation mechanism. Unlike T_1_ and T_2_, the dispersion effect can be used specifically by selecting and varying the SL amplitude [22, 23]. Here, primarily the amplitude of the SL pulse is decisive and the prevailing strength of the main magnetic field rather determines the achieved signal-to-noise-ratio (SNR). Thus, T_1ρ_ provides a more universal indicator and is only restricted by specific absorption rate (SAR) limitations. Recently, a study in diabetic monkeys was able to demonstrate T_1ρ_ dispersion within SAR limitations as a myocardial index for the early detection of diffuse fibrosis [8]. This study clearly demonstrates that the research of T_1ρ_ in animal models can provide important insights for specific development of clinical CMR sequences. Nevertheless, there are hardly any studies that deal with small animal models at high field strengths. One possible reason is the sensitivity of the required T_1ρ_ preparation to field inhomogeneities. Very recently, this has largely been compensated by optimization of the preparation modules [33–36]. However, critical physiological parameters (e.g. high respiratory and heart rates) still pose the major challenge for myocardial T_1ρ_ quantification in small animals.

Compared to humans, where the cardiac cycle has a duration of 600–1000 ms at rest, the cardiac cycle of mice is extremely short, commonly 100–140 ms [37]. As a result, complete acquisition in one cycle cannot be carried out for a single T_1ρ_ weighted image. In addition, the acquisition must take place in the desired cardiac phase (typically in the 20–30 ms time frame of the diastole) immediately after the SL preparation in order to receive pure T_1ρ_ contrast. Recovery of the longitudinal magnetization is necessary after each acquisition. Owing to the respiratory cycle (1000–2000 ms in mice), only one T_1ρ_ preparation and acquisition per respiratory cycle is possible under free-breathing. This leads to a tortuous prospective trigger procedure, which has to be completed by suitable respiratory gating. According to the literature [38], T_1ρ_ mapping has rarely been carried out in small animal studies [39–41]. Musthafa et al. [39] presented a study in a mouse infarction model, accounting a significant increase of T_1ρ_ at day 7 after infarction. The readout for data acquisition was based on a single Cartesian spin echo readout. This ensured high SNR but led to excessive measurement times of approximately 20 min for a single-slice map, although only four different T_1ρ_ weighted images were acquired. The same study also introduced an accelerated proof of concept for T_1ρ_ dispersion measurements using a gradient echo readout, with α = 15° and 8 acquisitions after each preparation [39]. Similarly accelerated quantification techniques were presented by Khan et al. [40] and Yla-Herttuala [41], with additional T_2_ and T_RAFFn_ preparations being considered. Finally, in [42], a significantly accelerated technique for myocardial T_1ρ_ quantification in mice was presented, in which radial gradient echo readouts and a KWIC filtered view sharing method were used.

In summary, gradient echo readouts have become the quasi standard for myocardial T_1ρ_ quantification in small animals due to shorter repetition times and faster k-space acquisition [39–42]. However, in the studies available to date, the influence of the readout on the T_1ρ_ relaxation pathway was neglected for the in vivo scenario. Due to incomplete T_1_ relaxation between successive preparations and readouts, the apparent T_1ρ_ values are affected by respiration, T_1_ and the sequence parameters. In this work we demonstrate that a value we refer to as T_1ρ_* is effectively observed under such in vivo conditions. Furthermore, we introduce a formal description of the T_1ρ_* relaxation pathway and thus enable the determination of the true T_1ρ_ value. The method is validated in phantom experiments and applied in vivo on mice. Moreover, due to retrospective applicability, our new approach enables the subsequent correction of prior study data.

### Theory

**Fig. 1:**
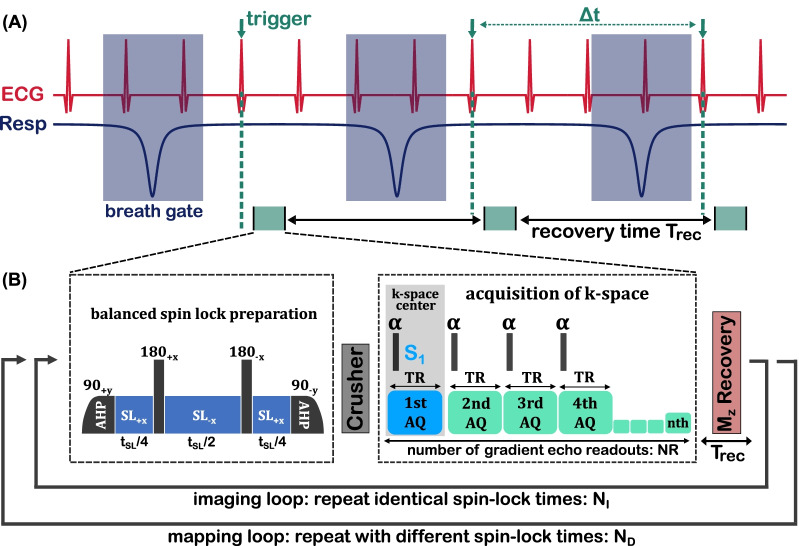
Sequence design for myocardial T_1ρ_ mapping in small animals. The high heart and respiratory rates require the use of prospective triggering in combination with breath gating. Usually a trigger on the R-wave and dynamic trigger delays are applied before the spin-lock (SL) preparation. The acquisition window in diastole is very short (≈20–30 ms), so that several readouts can only be carried out by using fast gradient echoes. To acquire a single T_1ρ_ weighted image, the experiment has to be repeated several times (N_I_). For T_1ρ_ mapping, imaging has to be repeated with different SL times (N_D_). The sequence parameters of the readout (T_rec_, TR, NR, α) have a decisive impact on the relaxation pathway of T_1ρ_. This is explained in Fig. 2 by considering the signal S_1_

Possible causes for T_1ρ_ quantification errors have already been discussed in several studies [22]. Compared to the influence of the preparatory pulse sequence [33–36], the readouts of accelerated T_1ρ_ techniques have not been examined as comprehensively [43–45]. In the context of CMR, multiple gradient echo readouts are usually acquired in the transient signal evolution towards steady-state after each preparation (Fig. 1) [39–42]. However, an analytical description of the signal equation was only derived for the case of NR = 1 acquisition after each preparation [43]. The general case of a multiple gradient echo readouts (NR ≥ 1) can be obtained by solving Bloch equations.

**Fig. 2:**
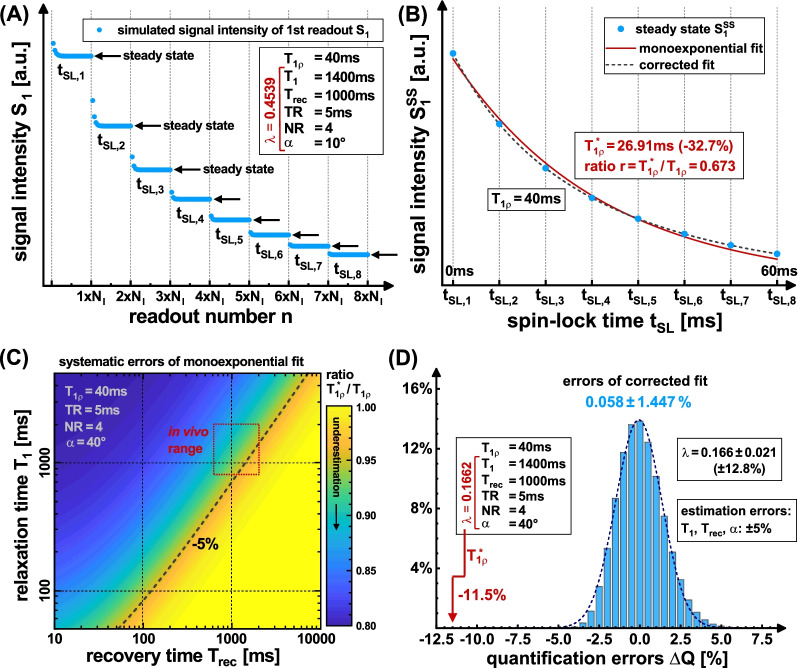
Simulation results of the investigation of quantification errors. The signal of the first acquisition S_1_ was calculated using Bloch simulations for realistic in vivo parameters (**A**). An individual steady state ${\mathrm{S}}_{1}^{\mathrm{SS}}$ is reached for each SL time. When using small flip angles, more repetitions are required for this. In **B** the values were fitted with the common monoexponential model. The relaxation time T_1ρ_* calculated in this way is systematically below the true T_1ρ_ value. The relationship between the systematic underestimation and the two parameters T_1_ and T_rec_ is shown in **C**. From this it can be seen that an incomplete spin recovery is the decisive point. A systematic error of − 2% to − 16% is to be expected in vivo. If the corrected fit is used (novel signal equation), errors only arise if incorrect sequence parameters are assumed (**D**). The error propagation is moderate here, since ± 5% estimation errors in the sequence parameters only lead to ± 1.447% errors in T_1ρ_

The transient evolution of longitudinal magnetization ${M}_{z}(n)$ in a RF pulse train can be described by a recursion:

1 $${M}_{z}[n]=\left\{\begin{array}{ll}{M}_{z,1},& n=1\\ { M}_{0}-\left({M}_{0}-{c\cdot M}_{z}\left[n-1\right]\right)\cdot {e}_{1},& n>1,\end{array}\right.$$

where ${e}_{1}=\mathrm{exp}[-TR/T1]$, $c=\mathrm{cos}[\alpha ]$ and n represents the n-th RF pulse (flip angle α). In addition, it has to be taken into account that the T_1ρ_ preparation is carried out before the first readout and that there is a recovery time delay T_rec_ to restore the longitudinal magnetization after the last readout (Fig. 1). As a result, after several repetitions of the experiment, ${M}_{z}$ reaches a steady state value ${M}_{z}^{SS}$ that is different for each readout (Fig. 2A). The steady state value for the first readout can be described by the following condition:

2 $${M}_{z}^{SS}\left[1\right]={M}_{z,1}=\left({ M}_{0}-\left({M}_{0}-{c\cdot M}_{z}\left[NR+1\right]\right)\cdot {e}_{2}\right)\cdot {e}_{3},$$

where ${e}_{2}=\mathrm{exp}[-{T}_{rec}/T1]$ and ${e}_{3}=\mathrm{exp}[-{t}_{SL}/{T}_{1\rho }]$. This recursion problem was solved in general (Additional file 1) using algebra software (Wolfram Mathematica 11.0, Wolfram Research, Champaign, IL, USA) and transformed into an explicit form:

3 $${M}_{z}^{SS}\left[1\right]={M}_{0}\frac{{e}_{3}}{1-{c}^{NR}{ e}_{1}^{NR}{ e}_{2}{ e}_{3}}\cdot \left[1-{e}_{1} {e}_{2}+{e}_{2} \sum_{k=1}^{NR-1}{c}^{k} \left({e}_{1}^{k}-{e}_{1}^{k+1}\right)\right].$$

The steady state solutions for the remaining readouts can be obtained by using Eq. (1) and the solution ${M}_{z}^{SS}\left[1\right]$ of the first readout. Thus a complete solution is given. The transverse magnetization can be further described by:

4 $${M}_{xy}^{SS}\left[n\right]={M}_{z}^{SS}\left[n\right]\cdot \mathrm{sin}\left(\alpha \right)\cdot {e}^{-\frac{TE}{{T}_{2}^{*}}.}$$

Since the T_1ρ_ contrast is primarily contained in the first readout, this is usually utilized for the acquisition of the k-space center. Therefore, we use the solution of the first readout as a novel signal equation for T_1ρ_ quantification:

5 $${S}_{1}^{SS}\left({t}_{SL}\right)={S}_{0} \cdot {\left({e}^{\frac{{t}_{SL}}{{T}_{1\rho }}}-\lambda \right)}^{-1},$$

where the following abbreviations were applied:

6 $$\lambda ={{\mathrm{c}}^{\mathrm{NR}}\cdot {\mathrm{e}}_{1}^{\mathrm{NR}}\cdot {\mathrm{e}}_{2}=\mathrm{cos}\left[\alpha \right]}^{NR}\cdot \mathrm{exp}\left[-\frac{NR\cdot TR}{{T}_{1}}\right]\cdot \mathrm{exp}\left[-\frac{{T}_{rec}}{{T}_{1}}\right],$$

and

7 $${S}_{0}={\Lambda \left({T}_{1},{T}_{rec},TR,NR,\alpha \right)\cdot \tilde{S }}_{0}.$$

8 $${\tilde{S }}_{0}\propto {M}_{0}\cdot \mathrm{ sin}\left(\alpha \right)\cdot {e}^{-\frac{TE}{{T}_{2}^{*}}}.$$

9 $$\Lambda \left({T}_{1},{T}_{rec},TR,NR,\alpha \right)=1-{e}_{1} {e}_{2}+{e}_{2} \sum_{k=1}^{NR-1}{c}^{k} \left({e}_{1}^{k}-{e}_{1}^{k+1}\right).$$

Here, we simplified the expression as much as possible by including all terms that do not explicitly depend on the SL time in ${S}_{0}$. In the case of NR = 1, our expression is equivalent to the signal equation in [43]. The influence of the readout can be summarized by the dimensionless parameter $\lambda$, which ranges from 0 to 1.

The signal evolution in Eq. (5) does not follow a monoexponential function. The expression shows that the relaxation pathway, which we refer to as T_1ρ_*, is influenced by T_1_ and the sequence parameters (recovery time T_rec_, repetition time TR, number of gradient echoes NR, flip angle α). If a simple monoexponential function.

10 $$S\left({t}_{SL}\right)={S}_{0}\cdot \mathrm{exp}\left[-{t}_{SL} /{T}_{1\rho }^{apparent}\right],$$

is used to fit T_1ρ_, significant quantification errors occur and systematically underestimated values ${T}_{1\rho }^{apparent}={T}_{1\rho }^{*}$ will be obtained (Fig. 2B).

## Methods

In the present work, the corrected T_1ρ_ quantification based on the new signal equation (Eq. 5) was compared with the conventional monoexponential model using simulations, as well as a phantom and an in vivo study.

First, the signal acquisition was simulated using Bloch equations in order to predict the quantification errors in several scenarios. Next, the correction technique was validated in phantom experiments. Finally, the method was tested in vivo for myocardial T_1ρ_ mapping under free-breathing conditions in mice. All measurements were performed on a 7.0 T small animal imaging system (Bruker BioSpec 70/30, Bruker BioSpin MRI GmbH, Ettlingen, Germany) with a maximum gradient field strength of 470 mT/m. A 35 mm quadrature transmit-receive birdcage was used for signal detection.

### Prediction of quantification errors based on Bloch simulations

In this section the influence of the sequence parameters on the T_1ρ_ quantification was examined. The steady-state magnetization ${M}_{1}^{SS}$ was numerically simulated by solving Bloch equations (Matlab R2018b, The MathWorks, Natick, MA, USA) with realistic parameters (T_1ρ_ = 40 ms, TR = 5 ms, NR = 4, α = 40°) for myocardial T_1ρ_ mapping in small animals. The effect of the recovery time T_rec_ and the relaxation time T_1_ was considered in detail, since these are variable in the in vivo experiment. For each combination of the parameters T_rec_ = 10 ms–10 s and T_1_ = 50 ms–5 s (250 × 250 logarithmically spaced values), the simulated signal was fitted (monoexponential model, Eq. 10) in order to map the relationship with the underestimated result T_1ρ_*. Furthermore, we investigated whether the relationship between the sequence parameter $\lambda$ and the ratio ${T}_{1\rho }^{*}/{T}_{1\rho }$ can be described using a lookup table (Additional file 1). Finally, we examined the susceptibility of corrected T_1ρ_ quantification based on the new signal equation (Eq. 5). For this it was assumed that the values T_1_, T_rec_ and α, which are required for the fit, are not exactly known. The simulation was based on normally distributed random numbers. In 10^6^ runs, 3 independent random numbers were generated for T_1_, T_rec_ and α using a standard deviation of ± 5%. T_1ρ_ was fitted for each triplet and the deviation from the true value T_1ρ_ = 40 ms was calculated.

### Phantom experiments

The phantom consisted of three cylindrical sample tubes (diameter 17 mm, length 120 mm) filled with different concentrations (15%, 20%, 25%) of bovine serum albumin (Sigma-Aldrich, St. Louis, Michigan, USA) diluted in demineralized water resulting in T_1ρ_ values in the typical range of biological tissue. In all experiments the T_1ρ_ preparation was performed by balanced spin-locking, which includes two adiabatic half-passage (AHP) excitation pulses, three continuous wave SL pulses using alternating phases and two opposite refocusing pulses for improved B_0_ and B_1_ compensation [36]. A Cartesian spoiled gradient echo readout was used for data acquisition. For the acquisition of the k-space center, the first of NR = 4 readouts was used after the preparation, while the k-space periphery was acquired with the remaining readouts (centric encoding). Furthermore, dummy runs were used to ensure that steady-state was reached before the first acquisition. For each map, 8 T_1ρ_ weighted images with t_SL_ = 4–95 ms (linearly spaced) were acquired.

In order to validate the corrected T_1ρ_ formalism, maps were acquired using 15 different magnetization recovery times T_rec_ = 0.5–10 s (logarithmically spaced). The T_1_ values for the correction were determined using an inversion recovery snapshot flash (IRSF) sequence [46]. The results of corrected T_1ρ_ were compared with the monoexponential model for each phantom in a circular region of interest (ROI). Here we also compared the R^2^ values (coefficient of determination) that were obtained for the different fit approaches. For corrected fitting the mean T_1_ values in the respective ROIs were used for each phantom. Apart from T_rec_, the remaining sequence parameters were kept constant: TR = 5 ms, TE = 2 ms, α = 40°, f_SL_ = 1500 Hz, matrix 128 × 96, fov 41.6 × 31.2 mm, slice thickness 1.5 mm.

### In vivo experiments

In order to validate the applicability of the corrected method in vivo, measurements were carried out in mice. Therefore, n = 14 healthy mice (C57BL/6, Naval Medical Research Institute, Charles River Laboratories, Willmington, Massachusetts, USA) were imaged in prone position. The animals were anesthetized with isoflurane inhalation (1.5–2 Vol. % in oxygen) and the body temperature was kept constant at 37 °C. For electrocardiographic (ECG) triggering two electrodes were attached to the forepaws of the mice and a pressure sensitive balloon placed on the abdominal wall was used for breath gating. All experimental procedures were in accordance with institutional guidelines and were approved by local authorities.

After a standard planning procedure, a midventricular short-axis imaging slice has been selected for the T_1ρ_ measurements. Compared to the phantom experiment, accelerated data acquisition based on a radial gradient echo readout and a KWIC-filtered (k-space weighted image contrast) view sharing method was used [42]. The acquisition window for the readout was positioned in end diastole using a variable trigger delay dependent on the respective SL preparation time. As shown in Fig. 1, one preparation followed by NR = 4 readouts was carried out in each breath cycle. The recovery time T_rec_ was thus determined by the respiratory cycle of the mice under free-breathing. In two animals, 10 T_1ρ_ maps were acquired in direct succession, with T_rec_ varying naturally. Here, a large variability in the respiratory cycle was observed in animal I (T_rec_≈1000–1700 ms), while only a slight drift occurred in animal II (T_rec_≈1300–1600 ms). Up to 5 repetitions of T_1ρ_ mapping were carried out in the remaining animals, which results in a total of N = 44 data sets with naturally varying T_rec_.

The results of the monoexponential fitting approach were compared to the results of the corrected fit in identical ROIs (left ventricular). It was further examined whether there is a correlation with the breath dependent recovery time. The two animals with 10 runs were evaluated individually. For corrected fitting we used the mean T_1_ values in the respective ROIs obtained by myocardial T_1_ quantification using a retrospectively triggered IRSF sequence [46]. For the global evaluation of the N = 44 data sets in n = 14 different animals, an estimated T_1_ value of 1400 ms was used for the correction. In order to determine the influence of the T_1_ estimate, the evaluation was repeated for ΔT_1_ =  ± 100 ms.

When performing the corrected T_1ρ_ fit, it was taken into account that T_rec_ was different for each T_1ρ_ weighting (Figs. 4A, 5A). The recovery time was calculated from the electronically recorded trigger time stamps:

11 $${T}_{rec}\left({t}_{SL}\right)=\overline{\Delta t }\left({t}_{SL}\right)-NR\cdot TR-{t}_{SL}-8ms,$$

where $\overline{\Delta t }$ is the averaged time difference between the time stamps for the respective SL time and 8 ms consists of constant timings of the SL preparation (excitation pulses, refocusing pulses, crusher gradients). Due to the dependency ${T}_{rec}\left({t}_{SL}\right)$ and potential breath drifts, it has to be considered that the parameter $\Lambda$ is variable (Eq. 9), whereby the fit function receives a prefactor depending on t_SL_.

The further in vivo sequence parameters were chosen similar to the phantom measurements: TR = 4.43 ms, TE = 1.85 ms, α = 40°, t_SL_ = 4, 12, 20, 28, 36, 44, 52, 60 ms, f_SL_ = 1500 Hz, matrix 128 × 128, fov 32 × 32 mm, slice thickness 1.5 mm.

### Simulation: detectability of increased T_1ρ_ in diseased tissue

According to literature, increased T_1ρ_ values are expected in diseased myocardial tissue. This has been shown in small animals in the infarcted mouse myocardium [39] and in fibrotic scars [40, 41] as well as in studies on patients [28, 29]. Based on the variations in respiration observed in the in vivo experiments (T_rec_ = 1.4 ± 0.19 s, drift = 0.7 ms/cycle), further simulations were performed to investigate the detectability of increased T_1ρ_ values in diseased tissue. For this purpose, baseline values of T_1ρ_ = 45 ms and T_1_ = 1400 ms (natural variation between different animals ± 1%) were assumed in healthy tissue. In diseased tissue, increased T_1ρ_ values of 1–5% were considered. For T_1_, variations in diseased tissue of – 10 to 10% were examined. The signal evolution during in vivo experiments was calculated by solving Bloch equations. For the sequence parameters, the setup of in vivo experiments described in the previous methods section were applied. The simulated signals were subsequently fitted using the uncorrected model and the new corrected model. Each simulation was repeated with 1000 variations of respiration and relaxation times, using normally distributed random numbers. The results of healthy and diseased tissue were tested for detectable significant difference by the means of t-test analysis. For the calculation of significance levels, 100 subgroups, each with 10 random simulated in vivo experiments, were evaluated, as this corresponds to a realistic study size. Based on this simulation framework, various scenarios were considered and tested to determine whether detection of diseased tissue is possible. The influence of the RF flip angle, the sensitivity to varying degrees of increased T_1ρ_, the influence of T_1_ variation, and the influence of the T_1_ values used for T_1ρ_ correction were investigated.

### Statistical analysis

Image reconstruction of radial CMR datasets was performed using the open-source MIRT Toolbox (Michigan Image Reconstruction Toolbox, University of Michigan, Ann Arbor, Michigan, USA). The T_1ρ_ weighted images were further processed using Matlab (Matlab R2018b, The MathWorks, Natick, Massachusetts, USA). Appropriate fitting-routines for the calculation of T_1ρ_ maps were implemented using a least square algorithm for minimum search of unconstrained multivariable functions for both the monoexponential model and the corrected model. All calculated T_1ρ_ maps were evaluated in the medial short-axis view, and global left ventricular mean values were determined in each case. Linear regression was performed for correlation analysis of measured relaxation times with recorded recovery times. The measure used was the Pearson correlation coefficient r (The MathWorks, Statistics and Machine Learning Toolbox). Continuous variables are expressed as mean ± standard deviation. Significance was based on a p-value of < 0.05, with p-values from simulations determined based on two-sample t-tests.

## Results

### Prediction of quantification errors based on Bloch simulations

The results of the simulation are shown in Fig. 2. In A-B the principle of the simulation for typical in vivo sequence parameters is presented schematically. In this specific case, the influence of the sequence parameters (λ = 0.4539) leads to an underestimation of the T_1ρ_ value by − 32.7%. If the flip angle of the readout is increased from 10° to 40° (λ: 0.4539 → 0.1662), the underestimation is only − 11.5%. For the case α = 40°, which we used in the measurements, Fig. 2C shows the underestimated value T_1ρ_* for varying T_1_ and T_rec_ values. Here it becomes clear that short T_rec_ and long T_1_ values cause a higher quantification error, since spin recovery between the T_1ρ_ preparations is incomplete. In the range of an in vivo experiment, a deviation of at best -2% and at worst − 16% can be expected. In the Additional file 1 we investigated how a connection between T_1ρ_, T_1ρ_* and the sequence parameter λ can be established, which enables a subsequent correction of systematic errors.

The use of the new signal equation does not lead to a quantification error in the simulation if the correct values of T_1_, T_rec_ and α are used for the fit. Since the exact knowledge of these parameters is unrealistic under experimental conditions, the error propagation was considered in a further simulation in Fig. 2D. A ± 5% deviation in T_1_, T_rec_ and α results in a ± 12.8% variation in the λ parameter. However, the T_1ρ_ quantification is comparatively stable with a mean error of (0.058 ± 1.447)%.

### Phantom experiments

**Fig. 3:**
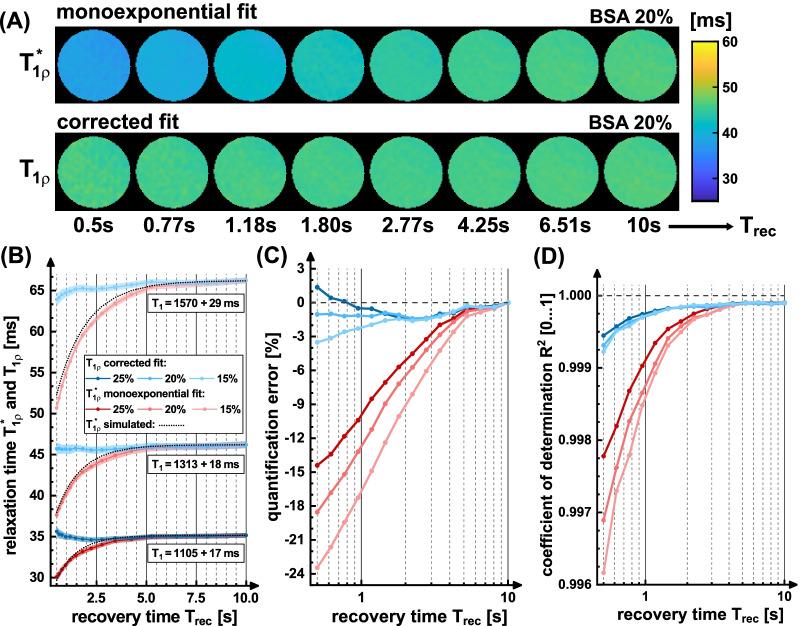
Results of the phantom experiments. In **A**, calculated relaxation time maps using the monoexponential model and the corrected model are compared. The increase in T_1ρ_* with T_rec_ can be seen visually. The measured T_1ρ_* values agree well with theoretically predicted values (**B**). The mean deviation from the prediction for the phantoms with increasing BSA concentration was 0.47%, 0.84% and 1.38%. The corrected fit, on the other hand, delivers nearly constant results. This is confirmed in the ROI based evaluation in **B** and **C**. The highest deviations arise in the phantom with the longest T_1_ relaxation time. Corrected fitting reduced the quantification error averaged over all measurements from -7.4% to -0.97%. The errors in the corrected fit could result from incorrect values of T_1_, T_rec_, or α. The R^2^ values are generally higher for the corrected fit (> 0.999). However, for monoexponential fitting, R^2^ values > 0.996 were achieved despite high quantification errors

The results of the phantom measurements are shown in Fig. 3. The impact of T_rec_ on T_1ρ_* can be visually recognized in the relaxation time maps (Fig. 3A). The corresponding maps of the corrected fit, however, indicates no trend. The ROI-based analysis (Fig. 3B–D) proofs that for all measured phantoms the T_1ρ_* value is significantly underestimated (− 14 to − 24%) at low T_rec_ and approaches the true T_1ρ_ value at high T_rec_. The corrected fit delivers constant values without an apparent trend. The phantom with the lowest BSA concentration shows a slight underestimation (− 3.5%) and the phantom with the highest concentration shows a slight overestimation (+ 1.4%). The different fit methods deliver nearly identical results for T_rec_ = 10 s (maximum deviation 0.05%). In Fig. 3B, theoretically predicted T_1ρ_* values were further added. These show good agreement with the experimental results and the mean deviation from the prediction is only 0.89%. The behavior of the R^2^ values is also noteworthy. Here the corrected fit achieves higher values R^2^ > 0.999. The monoexponential fit reaches at least R^2^ > 0.996, although large quantification errors were generated.

### In vivo experiments

**Fig. 4:**
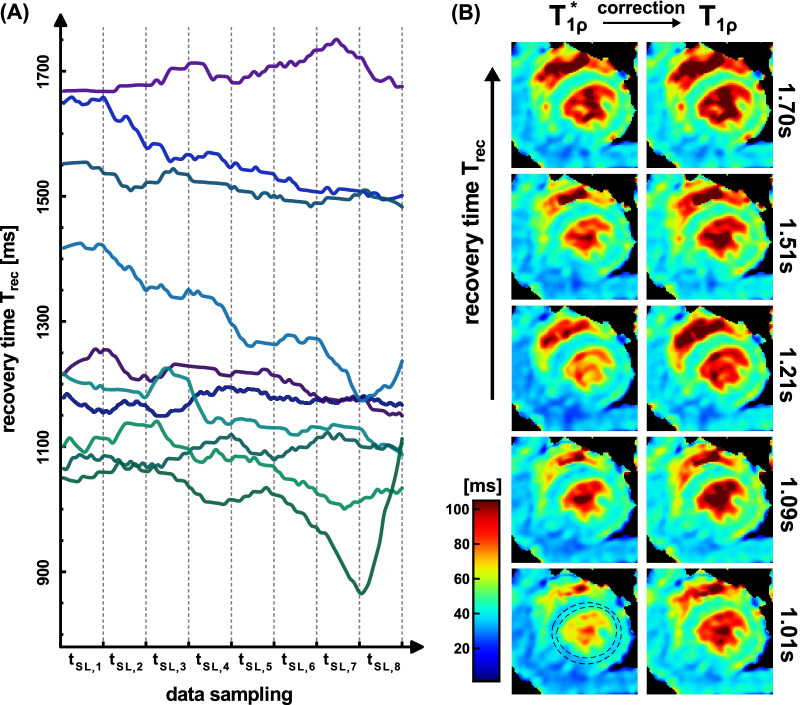
Results of the in vivo measurements at high T_rec_ variability. The recovery times recorded during the data acquisition are shown in **A**. The different colored curves show the individual T_rec_ times for 10 repetitions of the T_1ρ_ mapping sequence in animal I. In **B** the calculated relaxation time maps (short axis view, isotropic resolution 250 µm) for the monoexponential fit (left) and the corrected fit (right) are shown. 5 repetitions with different T_rec_ times are exemplary shown. The mean T_1_ value 1391 ± 34 ms in myocardial tissue was obtained by an inversion recovery snapshot flash (IRSF) sequence and used to calculate the corrected maps. The maps show distinct artifact formation, which is primarily due to the unsteady breathing. The region-of-interest (ROI) based correlation analysis with T_rec_ is shown in Fig. 6A

The results of the measurement at large T_rec_ variability are shown in Fig. 4. In the T_1ρ_* maps, a slight increase in the myocardial tissue can be visually identified with increasing T_rec_. Similar to the phantom experiment, this could not be assessed in the corrected maps. The ROI based evaluation (Fig. 6A) shows a significant positive correlation of T_1ρ_* with T_rec_ (Pearson correlation coefficient r = 0.684 with p < 0.05) and no significant correlation (r = 0.373) for the corrected fit. The normalized standard deviation of the 10 measurements was reduced from ± 4.8% to ± 2.0%. For the correction the value T_1_ = 1391 ± 34 ms was used.

**Fig. 5:**
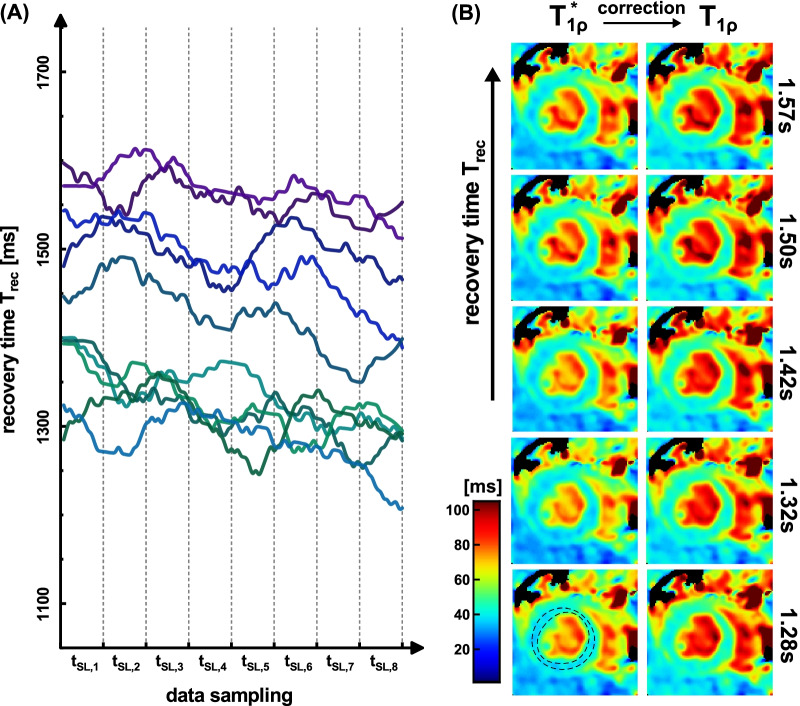
Results of the in vivo measurements at low T_rec_ variability. The recovery times recorded during the data acquisition are shown in **A**. The different colored curves show the individual T_rec_ times for 10 repetitions of the T_1ρ_ mapping sequence in animal II. In **B** the calculated relaxation time maps (short axis view, isotropic resolution 250 µm) for the monoexponential fit (left) and the corrected fit (right) are shown. 5 repetitions with different T_rec_ times are exemplary shown. The mean T_1_ value 1342 ± 44 ms in myocardial tissue was obtained by an IRSF sequence and used to calculate the corrected maps. A high image quality and less streaking is perceptible due to reduced motion issues. The ROI based correlation analysis with T_rec_ is shown in Fig. 6B

**Fig. 6:**
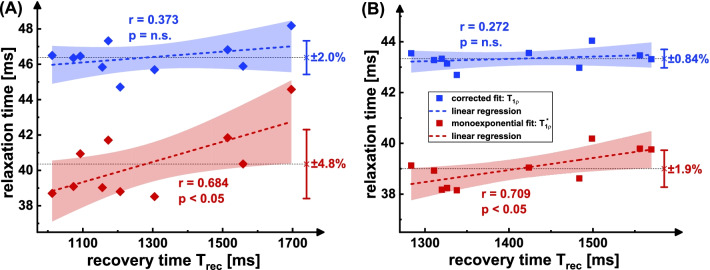
Correlation analysis of the measured relaxation times with T_rec_. The T_1ρ_ and T_1ρ_* values are the mean values within the left ventricular ROIs (Figs. 4B, 5B). The T_rec_ values correspond to the averaged recovery times during the corresponding individual measurements (Figs. 4A, 5A). **A** Shows the results with high and (**B**) shows the results with low T_rec_ variability. In both cases there is a significant positive correlation (r = 0.684, r = 0.709, p < 0.05) for uncorrected monoexponential fitting and no significant correlation (r = 0.373, r = 0.272) for corrected fitting. The plots also show the 95% confidence intervals (light red/blue areas) and the respective mean values and standard deviations as error bars in both cases

The results at low T_rec_ variability are shown in Fig. 5. Here, a higher image quality with reduced streaking is observable. The impact of T_rec_ can hardly be assessed visually. In the ROI based evaluation (Fig. 6B), T_1ρ_* shows a significant correlation with T_rec_ (r = 0.709 with p < 0.05). The corrected T_1ρ_ values show no significant correlation (r = 0.272). The normalized standard deviation was reduced from ± 1.9% to ± 0.84%. Despite a slight T_rec_ variation, the T_1ρ_* value was underestimated by an average of − 9.9% for monoexponential fitting. For the correction the value T_1_ = 1342 ± 44 ms was used.

**Fig. 7:**
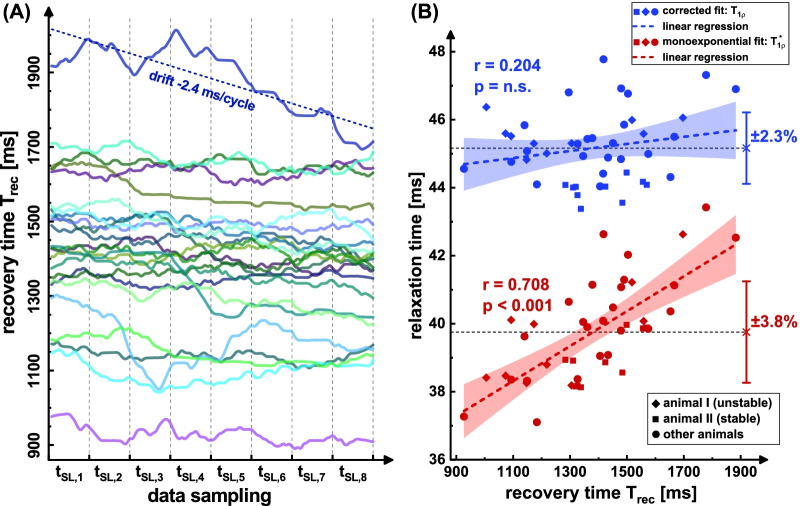
Correlation analysis of the measured relaxation times with T_rec_ for n = 14 different animals and N = 44 individual measurements. The recovery times recorded during the data acquisition are shown in **A** for the remaining animals. The evaluation was carried out analogously to animal I and animal II (Figs. 4, 5, 6). Here, corrections were made for all animals with the T_1_ estimate of 1400 ms. In **B** a significant positive correlation for uncorrected fitting (r = 0.708, p < 0.001) and no significant correlation (r = 0.204) for corrected fitting was identified. The plots also show the 95% confidence intervals (light red/blue areas) and the respective mean values and standard deviations as error bars in both cases. Since comparisons were made between different animals, the variation in relaxation times is higher here than, for example, in the single study of animal II

Likewise, the evaluation of different animals (Fig. 7) shows significant positive correlation (r = 0.708, p < 0.001) with the recovery time for the uncorrected T_1ρ_*. Using the T_1ρ_ correction approach, only a slight correlation (r = 0.204, not significant) remains. The normalized standard deviation was reduced from ± 3.8% to ± 2.3%, which corresponds to an improvement of 39%. The choice of the T_1_ estimate (ΔT_1_ =  ± 100 ms) shows only a slight influence on the corrected values. A variation of the mean value of 45.2 ± 0.5 ms was found (± 1.1%) and the correlation with T_rec_ was hardly changed (r = 0.191–0.222).

### Simulation: detectability of increased T_1ρ_ in diseased tissue

**Fig. 8:**
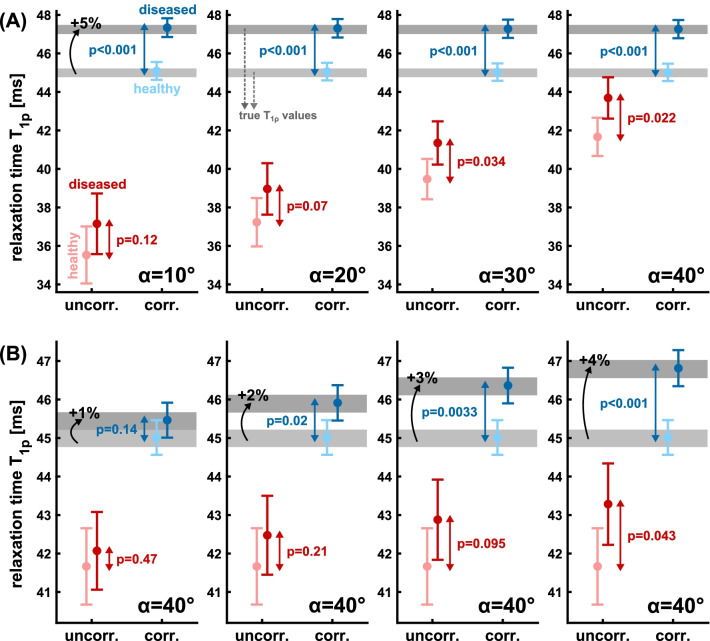
Simulation of in vivo experiments for the detectability of diseased tissue. The gray areas illustrate the true T_1ρ_ values for healthy (light gray) and diseased tissue (dark gray). A natural variation of ± 1% was assumed in the simulation for the N = 1000 random experiments. The uncorrected fit results (red) and the corrected fit results (blue) were compared. In **A**, the impact of radiofrequency (RF) flip angle choice (α = 10–40°) on quantification accuracy and significance levels at 5% increased T_1ρ_ in diseased tissue was investigated. In **B** the sensitivity of detectability for increased T_1ρ_ in the range 1–4% was investigated for α = 40°. The corrected fit provides consistent results for all simulated RF flip angles (p < 0.001) and improved detection sensitivity for increased T_1ρ_ (p < 0.05 for + 2%)

**Fig. 9:**
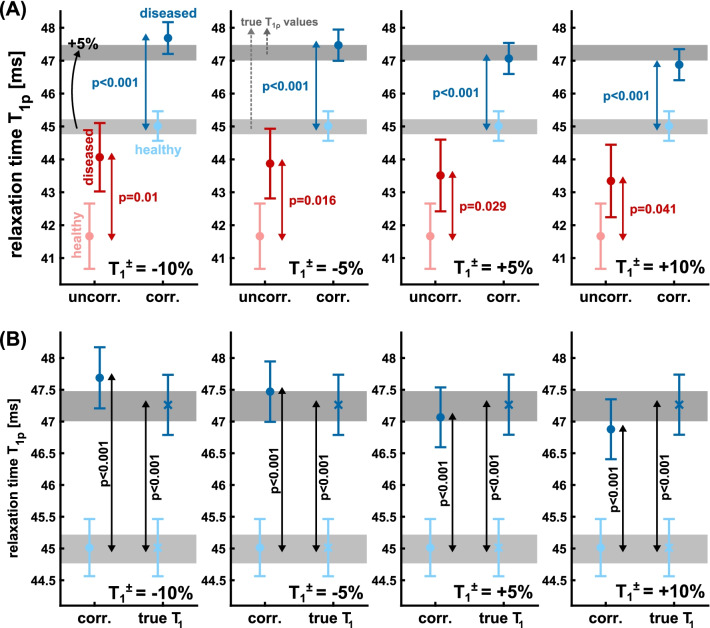
Simulation of in vivo experiments for the detectability of diseased tissue. The gray areas illustrate the true T_1ρ_ values for healthy (light gray) and diseased tissue (dark gray). A natural variation of ± 1% was assumed in the simulation for the N = 1000 random experiments. For the RF flip angle α = 40° was used. The impact of varying T_1_ values in diseased tissue on T_1ρ_ quantification and detectability was investigated. A 5% increased T_1ρ_ in diseased tissue was considered. In **A**, the uncorrected fit results (red) and the corrected fit results (blue) were compared. Here, the correction was performed using the baseline T_1_ value (1400 ms) for both healthy and diseased tissue. In (**B**), the corrected fit using the true T_1_ values of healthy and diseased tissue was supplemented. The results show significantly improved detectability based on the corrected fit (**A**). Using true T_1_ values instead of baseline values, the quantification accuracy can be slightly increased (**B**)

The simulation results of t-test analyses for the detectability of increased T_1ρ_ in diseased tissue are presented in Figs. 8 and 9. Figure 8A shows the influence of the RF flip angle (α = 10°–40°) at 5% increased T_1ρ_. The uncorrected fit provides severely underestimated T_1ρ_ values for both healthy and diseased tissue. This underestimation is smaller for larger flip angles. The corrected fit provides consistent results and most importantly allows a significant detection of diseased tissue (p < 0.001) for all simulated flip angles. The uncorrected fit provides a significance p < 0.05 only for α = 40°. Figure 8B illustrates the sensitivity to increased T_1ρ_ values (1–4%) for α = 40°. Also in this scenario, the corrected fit provides improved detection of diseased tissue. Here, a T_1ρ_ increase of 2% can be detected significantly (p < 0.05). Figure 9A shows the results at 5% increased T_1ρ_ with additionally altered T_1_ (− 10 to 10%) in the diseased tissue. However, the correction was only performed with the mean baseline value T_1_ = 1400 ms. This scenario indicates that reduced T_1_ in diseased tissue simplifies detection for the uncorrected fit. Increased T_1_ leads to significantly poorer detectability. The corrected fit allows constant detectability for – 10 to 10% (p < 0.001). Nevertheless, it is evident that altered T_1_ values affect quantification, leading to overestimation in the case of reduced T_1_ and underestimation in the case of increased T_1_ in diseased tissue. Figure 9B presents data, in which the T_1ρ_ correction was performed using the true T_1_ values of diseased tissue, representing a pixel-by-pixel correction with a T_1_ map. Here, the underestimation and overestimation of T_1ρ_ values in diseased tissue (Fig. 9A) was not observed. Pixel-wise correction with true T_1_ values is thus the ideal case for correction. However, a correction with baseline T_1_ values also allows reliable detectability with p < 0.001.

## Discussion

In the present study, a new formalism for corrected T_1ρ_ quantification was presented. The formalism is aimed specifically at time-critical measurements such as myocardial T_1ρ_ mapping in small animals. The method was validated in simulations and successfully applied in phantom experiments and in vivo on mice. Here, systematic errors and correlation with respiration were significantly reduced, improving the investigation of T_1ρ_ in small animal models.

The simulation results show that the monoexponential model can lead to high systematic errors and the effective observation of an underestimated relaxation time T_1ρ_*. Here, the decisive factor is the combination of T_1_ and T_rec_, as this determines the spin recovery. However, the flip angle α and the number of readouts per preparation NR are also important factors determining spin history. By choosing high flip angles and multiple readouts, the signal equation approaches the monoexponential model (${\mathrm{cos}\left[\alpha \right]}^{NR}\to 0$). Nevertheless, this was not the case in studies that previously dealt with T_1ρ_ quantification in small animals [39–42]. Given the parameters in other studies (e.g. NR = 8 and α = 15°) the simulations show that a systematic underestimation of at least − 11% is expected even with T_rec_ = 2 s. In [42] and in the present work a readout with NR = 4 and α = 40° was used. Here the systematic underestimation is at least − 5%. These estimates can be calculated from the results of our simulation, which also enables subsequent correction of study results (Additional file 1). Yet, the simulations also show that the corrected fit leads to quantification errors when assuming incorrect T_1_, T_rec_ and α values. We were able to prove that the error propagation is moderate and 5% variations of the fit constants lead to only 1.4% errors in T_1ρ_.

The corrected fitting procedure was validated in phantom experiments. The behavior of the T_1ρ_* quantification can be explained by the increasing spin recovery with larger T_rec_ and thus agrees well with the predictions of the simulation. The remaining errors of the corrected fit can result from an over-/underestimation of the respective T_1_ values and the effective flip angle. However, we were able to reduce the quantification error from − 7.4% to − 0.97% (averaged over all experiments). It has also been observed that monoexponential fitting produces high R^2^ values even with high quantification errors, which can falsely give the impression of successful quantification. The experiment further shows that the new signal equation enables particularly fast T_1ρ_ quantifications. The fastest acquisition was 1.6 min with an average error of − 1.1%. However, further acceleration is possible by using a larger NR, whereby the SNR is a limiting factor.

The in vivo measurements of the first animal with high T_rec_ variability demonstrate the problem of monoexponential fitting in practical use. Due to the fast changes of breath and heart rate during data sampling, artifact formation was observed in all images (e.g. streaking). For the measurements, which represent a worst-case scenario, a range of ${\overline{T} }_{1\rho }^{*}=38.5-44.6ms$ was observed. By corrected fitting, the normalized standard deviation of the 10 individual measurements could be reduced significantly from ± 4.8% to ± 2.0% (${\overline{T} }_{1\rho }=44.7-48.2ms$). The measurements of the second animal with only small T_rec_ variability correspond to the desired ideal case of an in vivo experiment under free-breathing. But even in this case the T_rec_ correlation was still observed for monoexponential fitting (r = 0.709), which shows that the correction is generally necessary. A higher stability of breathing is impossible to warrant under free-breathing and could only be guaranteed by assisted ventilation. For the interpretation of the correlation data it has to be considered that the T_rec_ values used are only averaged values during the entire data acquisition. In the case of strong drifts during the acquisition (Fig. 7A), the abscissa position cannot be precisely assigned in the correlation analysis. This could be one reason why only values r < 0.8 were found. However, the drift effect was taken into account for the corrected T_1ρ_ fit algorithm. Here, it was considered that T_rec_ can be different for each T_1ρ_ weighting (Eqs. 9 and 11). In the Additional file 1 we demonstrate that the drift effect can systematically impact the quantification. If the physiological data of respiration have not been recorded, an approximate correction can still be made.

The experiments carried out in this study show that the use of the new signal equation enables increased quantification accuracy. For this, however, the sequence parameters (TR, NR, T_rec_ and α) and the relaxation time T_1_ must be known. For protocols not containing T_1_ mapping, this can lead to limitations, since the correction has to be performed using estimated values. Nevertheless, as has been shown, even the use of a constant literature value or an estimate (e.g. T_1_ = 1400 ms in myocardial tissue) can reduce systematic errors and the T_rec_ correlation. The simulations performed on the detectability of increased T_1ρ_ in diseased tissues show an improvement over the uncorrected model in all scenarios considered. A significance of at least p < 0.05 can be expected from 2% increased T_1ρ_. The influence of the T_1_ values used for correction was also examined. Correction with true T_1_ values (e.g. pixel-by-pixel correction with a T_1_ map) is preferable and provides higher quantification accuracy. However, detectability of increased T_1ρ_ is also possible with baseline values and the quantification error for a – 10 to  + 10% systematic error in T_1_ estimation is only + 0.90 to − 0.82%.

Besides the approach presented in this study, several methods for rapid T_1ρ_ quantification have been published that minimize the influence of the readout sequence. In [44] a 3D T_1ρ_ quantification technique based on steady-state spoiled gradient echoes was introduced. This method provides significant acceleration in data acquisition while reducing SAR and has been tested for cartilage imaging. In [47] an accelerated 3D T_1ρ_ sequence based on balanced steady-state free precession readouts and a transient signal decay k-space filter was presented and tested in the knee joint and the lower lumbar spine. For myocardial T_1ρ_ quantification in humans, a sequence using transient signal stabilization between spin-lock and spatial encoding was presented [48]. In addition, a motion correction was used which exploits that each T_1ρ_ weighted image is acquired in one single heartbeat and the entire T_1ρ_ map is generated in a single breath hold [49] provided further acceleration for 3D myocardial T_1ρ_ quantification by adopting multi-coil compressed sensing. In [28] an additional magnetization reset pulse was applied after the cardiac trigger, since heart rate variability during the scan was found to impact T_1ρ_ accuracy in humans. Some of these approaches, such as acceleration by multi-coil compressed sensing or motion correction, could also be partially transferred to myocardial T_1ρ_ quantification in small animals. However, the basic structure of our pulse sequence is severely limited due to the specific physiological conditions in mice. For this reason, the method and correction presented in this work can be considered unique for experimental conditions in small animals. The use of transient signal stabilization after SL preparation [48] or the execution of a magnetization reset between cardiac trigger and SL [28] is not possible due to the high heart rate in mice. Yet, a reset after the last readout would be possible. A disadvantage of this technique is that if the recovery time drifts within the measurement, the correction would still be required. Furthermore, this method leads to an increase in SAR, whereas the SNR would be decreased.

In the present work a new signal equation was presented which can generally be applied for sequences using magnetization preparations and gradient echo readouts. Applications for the quantification of T_2_ or T_RAFFn_ are also feasible with only the T_1ρ_ relaxation term having to be replaced. Moreover, the results of this work can be used for the design of further improved sequences showing the least possible susceptibility of T_1ρ_ quantification to readout parameters and in vivo conditions. A key for this is the optimization of the sequence parameter λ, adapted to the respective application. In this context, work is in progress to develop a sequence based on variable flip angle trains. For this purpose, concepts from T_1_ and T_2_ quantification for the correction of flip angle profiles could be transferred [50]. Besides, the relaxation pathway of T_1ρ_* could be adapted specifically for the simultaneous measurement of T_1_ and T_1ρ_ in the context of magnetic resonance fingerprinting [51–53]. Since the relaxation path is completely analytically described within the derived formalism, sequences can be designed with a targeted T_1_ weighting and thus T_1_ and the corrected T_1ρ_ could be determined simultaneously.

## Conclusion

In conclusion, our corrected quantification method provides fast and accurate T_1ρ_ mapping in small animals during free-breathing. Our new technique thus offers reliable assessment of myocardial diseases. Pathologies that cause variation in heart or breathing rates do not lead to systematic misinterpretations and could therefore be examined more precisely. Consistent application of the correction can therefore be used for greater comparability or for subsequent correction of prior study results. In addition, the formalism is universally applicable and can also be used for sequence optimization, the correction of T_2_ or T_RAFFn_ and potentially for simultaneous multiparameter quantification.

## Supplementary Information

**Additional file 1:** Additional information, simulation data and experimental results.

## Data Availability

The datasets used and/or analyzed during the current study are available from the corresponding author on reasonable request.

## Abbreviations

AHP: Adiabatic half-passage
CMR: Cardiovascular magnetic resonance
ECG: Electrocardiogram
IRSF: Inversion recovery snapshot flash
LGE: Late gadolinium enhancement
MI: Myocardial infarction
RF: Radiofrequency
ROI: Region of interest
SAR: Specific absorption rate
SL: Spin-lock
SNR: Signal-to-noise ratio
