# Supplementary material for: Quantification correction for free-breathing myocardial T1ρ mapping in mice using a recursively derived description of a T1ρ* relaxation pathway
Source: J Cardiovasc Magn Reson. 2022 May 9;24:30. doi: 10.1186/s12968-022-00864-2 (PMC9082875; doi:10.1186/s12968-022-00864-2)
Supplement: Supplementary file 1 — Additional file 1: Additional information, simulation data and experimental results. [file 12968_2022_864_MOESM1_ESM.pdf]

SUPPLEMENTARY MATERIAL

Quantification correction for free-breathing myocardial $T_{1\rho}$ mapping in mice using a recursively derived description of a $T_{1\rho}^*$ relaxation pathway

Maximilian Gram[1,2], Daniel Gensler[1,3], Petra Albertova[2], Fabian Tobias Gutjahr[2,3], Kolja Lau[1], Paula-Anahi Arias-Loza[3,4], Peter Michael Jakob[2], and Peter Nordbeck[1,3]

[1]*Department of Internal Medicine I, University Hospital Würzburg, Würzburg, Germany*
[2]*Experimental Physics 5, University of Würzburg, Würzburg, Germany*
[3]*Comprehensive Heart Failure Center (CHFC), University Hospital Würzburg, Würzburg, Germany*
[4]*Department of Nuclear Medicine, University Hospital Würzburg, Würzburg, Germany*

Address for correspondence: Dr. Peter Nordbeck M.D., University Hospital Würzburg, Department of Internal Medicine I, Oberdürrbacher Str. 6, Würzburg, DE D-97080, nordbeck_p@ukw.de

Attached Online Files: Mathematica Notebook (Wolfram Research, Inc.)
.pdf, .nb, .cdf

Online Figures
Fig 1, Fig 2, Fig 3

Approximation of lookup table data: $T_{1\rho}^*/T_{1\rho}(\lambda)$

In the present work, a novel signal equation for $T_{1\rho}$ quantification was derived. The derivation, which was based on the solution of a recursion problem, can be followed step-by-step in the attached Online Files (Mathematica Notebook).
It has been found that the deviation from the simple monoexponential model can be expressed by a single global sequence parameter λ (dimensionless $[0\ldots1]$). Knowing λ, $T_{1\rho}$ can be determined with the corrected fit. If the monoexponential model is used instead, systematically smaller values of $T_{1\rho}^*$ are obtained. Online Figure 1 shows the ratio $r_\rho^* = T_{1\rho}^*/T_{1\rho}$ as a function of λ from the data of a lookup table. Here it can be seen that small values λ lead to small quantification errors, while large values lead to high systematic deviations. The $r_\rho^*(\lambda)$ relationship was numerically described by a polynomial fit. The results of this fit can be used to determine the approximated value $T_{1\rho}^{\text{approx}}$ for a measured $T_{1\rho}^*$ at an arbitrary λ.

$$T_{1\rho}^{\text{approx}} = \frac{T_{1\rho}^*}{r_\rho^*(\lambda)} \quad \textbf{Online Equation 1}$$

Subsequent correction of study results

The approximated $T_{1\rho}$ correction was exemplarily applied to myocardial $T_{1\rho}$ mapping in mice. Here, the results of $T_{1\rho}^{*}$ and $T_{1\rho}$ (already presented in Figure 7) were supplemented with subsequently corrected $T_{1\rho}^{\text{approx}}$ values (Online Figure 2). For each data point, the $T_{1\rho}^{*}$ value (monoexponential fit) was used to calculate an approximated $T_{1\rho}^{\text{approx}}$ value based on the corresponding λ and Online Equation 1. Here, λ was calculated using the averaged T_{rec} during the complete data acquisition. Thus, the corrected fitting approach is not necessary and the approximated $T_{1\rho}^{\text{approx}}$ values can be subsequently calculated from the underestimated $T_{1\rho}^{*}$ values. This technique yields values that show good agreement with the corrected $T_{1\rho}$ values. However, the standard deviation of $T_{1\rho}^{\text{approx}}$ (±2.8%) is slightly higher than the standard deviation of the corrected values (±2.3%).

Online Figure 3 examines whether there is a correlation between the discrepancy ($T_{1\rho}^{\text{approx}}$ vs $T_{1\rho}$) and respiration. It can be seen that there is no correlation with T_{rec} itself (Online Figure 3A, $r = 0.068$, not significant). However, we found a positive correlation with the drift of recovery time during data acquisition (Online Figure 3B, $r = 0.865$, $p < 0.001$). The drift values were in a range of -2. . . 1ms/cycle for the N=44 measurements. The discrepancy ($T_{1\rho}^{\text{approx}}$ vs $T_{1\rho}$) was between -5. . . 3%. If there is no drift, $T_{1\rho}^{\text{approx}} \approx T_{1\rho}$. This result indicates that approximated $T_{1\rho}$ calculation is in principle an improvement over $T_{1\rho}^{*}$. However, the results can be systematically influenced by a drift of the respiration during the measurement. Thus, a fit with the new signal equation (Equation 5) considering the respiratory drift (Equation 11) is preferable, provided that the physiological data were recorded completely. For an approximated correction (based on an averaged T_{rec}), we have provided a computable interactive document (Online File: .cdf).

Online Figures

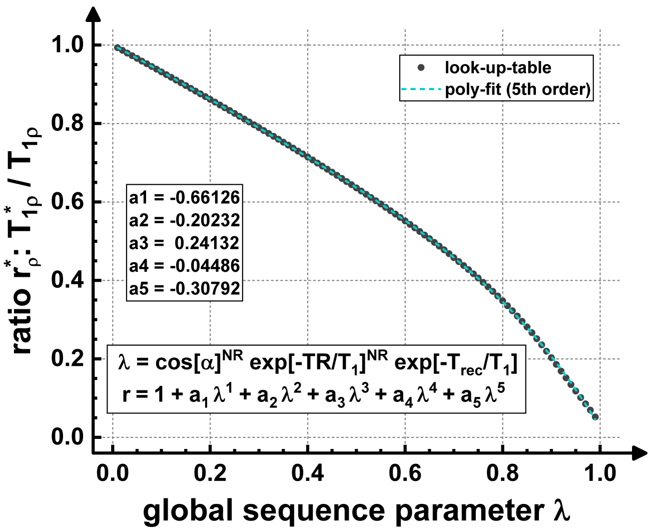

Online Figure 1: The ratio $r_{\rho}^{*} = T_{1\rho}^{*}/T_{1\rho}$ was calculated as a function of the global sequence parameter λ. A lookup table was calculated based on 100 different values of λ and for 100 different $T_{1\rho}$ values in the range 10...200ms. No direct dependence of the ratio on $T_{1\rho}$ itself could be found. The data of the lookup table were approximated with a 5th order polynomial fit ($R^2 > 0.999$). The results of this approximation were used for subsequent correction of study results (Online Figure 2).

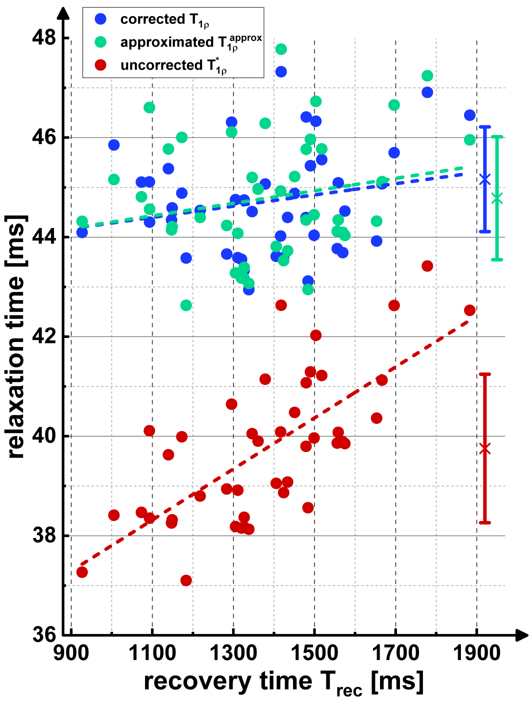

Online Figure 2: Comparison of myocardial $T_{1\rho}$ mapping in mice based on monoexponential fitting, corrected fitting and the subsequent correction using the approximated ratio $r^*_\rho(\lambda)$. The approximated values clearly separate from $T^*_{1\rho}$ and show good agreement with the corrected $T_{1\rho}$ values fitted by the novel signal equation 5. However, a slightly higher variation is observed (±2.8% vs ±2.3%) for the approximation. The correlation with T_{rec} is not significant (r=0.068).

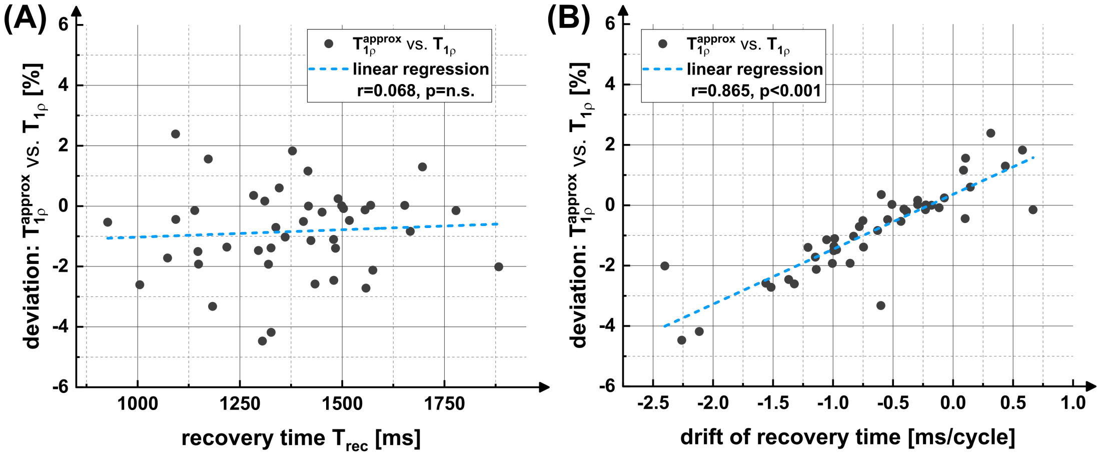

Online Figure 3: Correlation of the discrepancy ($T^{\text{approx}}_{1\rho}$ vs $T_{1\rho}$) with the recovery time T_{rec} (A) and the drift of recovery time (B). There is no correlation with T_{rec} itself. However, with the drift a clear positive correlation can be proven. The drift values were determined from the slope of the linear regression of the T_{rec} courses. The units of the drift values (ms/cycle) indicates how large the change in T_{rec} is per respiratory cycle. In each respiratory cycle, one spin-lock preparation was performed followed by NR=4 readouts in the diastole. Since first short and then long spin-lock times were sampled and the recovery time also depends on the spin-lock time (Equation 11), the drift values tend to negative values.
